# Majority of the Dutch Primary Dementia Care Networks Do Not Include Allied Health Professionals

**DOI:** 10.2147/JMDH.S511932

**Published:** 2025-05-02

**Authors:** Tijmen Geurts, Dorien L Oostra, Marcel G M Olde Rikkert, Minke S Nieuwboer, Marieke Perry

**Affiliations:** 1 Department of Geriatric Medicine, Radboud Research Institute, Radboud University Medical Center, Nijmegen, the Netherlands; 2 Radboudumc Alzheimer Center, Radboud University Medical Center, Nijmegen, the Netherlands; 3 HAN University of Applied Sciences, Academy of Health and Vitality, Nijmegen, the Netherlands; 4 Department of Primary and Community Care, Radboud University Medical Center, Nijmegen, the Netherlands

**Keywords:** allied healthcare, primary healthcare, interprofessional, dementia

## Abstract

**Background:**

Allied health professionals can contribute to better quality of life of people with dementia. However, it is unclear whether DementiaNet networks effectively integrate their expertise. We aim to describe the extent of allied health involvement in DementiaNet networks.

**Methods:**

Between 2015 and 2021, 35 currently active primary care networks were formed. During this period, logs of the network’s composition were kept and used to describe allied health involvement.

**Results:**

Ten networks included at least one allied health professional at the start of the project, which increased to 17 networks at follow-up. Networks with allied health professionals were larger than average and predominantly situated in (sub)urban areas.

**Conclusion:**

Less than half of the DementiaNet networks included allied health professionals at follow-up. The reasons for this are unknown. Therefore, exploration of barriers and facilitators for allied health involvement is necessary to engage allied health professionals and improve interprofessional collaboration.

## Introduction

Many primary care professionals are involved in caring for home-dwelling older adults with dementia. The complexity of older adult care has increased the necessity of collaborative initiatives.^1^ In the Dutch healthcare system, most allied health professionals are active in primary care as independent caregivers, and competition between healthcare professionals is common. With DementiaNet, a 2-year integrated care program, we facilitated collaboration between primary healthcare professionals by forming local care networks.^2^ During the project, network participants learned to work according to an interprofessional approach. As a result, network collaboration and quality of care improved during the project.^3^

In these networks, involvement of allied health professionals was stimulated, as they can contribute to improved quality of life and daily functioning of people with dementia.^4–7^ However, whether their expertise was effectively included in DementiaNet networks is unclear. We aim to describe to which extent allied health professionals are involved in these networks at baseline and follow-up of the DementiaNet program.

## Methods

During the DementiaNet project, between 2015 and 2021, 35 currently active primary care networks were formed.^3^ These networks comprise primary healthcare professionals such as general practitioners, community nurses, and case managers sharing a joint caseload. Networks were supported by the research team for the first two years of the program. During this period, network leaders were selected and professionals were trained to focus on areas of improvement according to Plan-Do-Check-Act (PDCA)-cycles.^8^ After two years, the support from the research team stopped, but networks continued while maintaining contact with the research team. During and after the project, networks were composed based on local preferences and professional availability. Networks decided for themselves who they wanted to include and did not have to report reasons for why they changed in composition.

Logs of the network composition were kept from the start of each network until the end of the DementiaNet project in 2021. Using this data, we described network characteristics and the involvement of various allied health disciplines at both the start of the project and up to six years of follow-up.

## Results

### Allied Health Professional Involvement at Start

Ten networks (28.6%) included allied health professionals at the start of their trajectory, of which three included an occupational or physiotherapist as a network leader. Nine of these ten networks included two or more allied health professionals from at least two disciplines. Physiotherapists were involved in all ten networks, while occupational therapists were involved in nine. Speech-language pathologists were involved in three networks, and dieticians in two networks.

The ten networks with allied health professionals were larger compared to the average number of network participants (M=14.4 versus 10.3, Mdn=16 versus 8) and predominantly (sub)urban (80%). Networks without allied health involvement (N=25) were predominantly rural (N=16, 64%).

### Allied Health Professional Involvement at Follow-up

At the end of DementiaNet project, 17 networks (48.6%) included allied health professionals. Out of the ten networks that already included allied health professionals at the start of the DementiaNet trajectory, three changed their allied health composition. One network added two physiotherapists, one network added a dietician, and one network added a physiotherapist, but the psychologist and speech-language pathologist stopped participating in the latter.

Of networks with allied health involvement, 11 out of 17 (64.7%) networks were situated in a (sub)urban area, while six out of 18 (33%) networks without allied health involvement were (sub)urban.

## Discussion

Allied health disciplines were involved in 17 of the 35 DementiaNet networks at follow-up. This suggests that some primary care networks might not see the additional value of allied health disciplines in improving their joint dementia care. Lacking robust evidence for allied health benefits for people with dementia could be an explanation.^9^,^10^ Networks might not have focused on areas of improvement that necessitate allied health involvement. Another reason might be that some of these networks did not want to add new members since building familiarity and trust with new network members has been shown to take time and effort, especially for large networks.^11^ Still, the number of networks with allied health professionals did increase over time, which suggests that the DementiaNet program promoted allied health involvement in some way.

For allied health professionals, financial constraints and an increasing workload may play a role. Many allied health professionals already participate in larger scale networks with a focus on other chronic diseases, such as ParkinsonNet^12^ or COPDnet.^13^ This could explain why they rarely act as network leaders, along with allied health professionals often being considered specialized disciplines without the final responsibility for the care of a patient with dementia.

When allied health professionals are involved, they are mainly occupational- and/or physiotherapists. An occupational therapist’s expertise is broader and less defined than a physiotherapist’s. However, due to the widespread evidence-based COTiD-training program,^14^ their expertise in dementia care is increasingly well known. In comparison with these two disciplines, dieticians are less involved, indicating that there is still unawareness of malnutrition as a problem in home-dwelling older adults.^15^ Other allied health disciplines such as podiatrists and speech-language pathologists rarely take part in the networks. These disciplines are fewer in number, but in addition, networks may have the perception that their work is too specific to consider their inclusion.

DementiaNet networks with allied health involvement are often located in (sub)urban regions. It might be that allied health professionals are dealing with more competition in urban areas, necessitating promoting their practices via a network. In rural areas, allied health practices are somewhat more dispersed. Ties with nearby allied health professionals might already be established and may not need to be formalized in a network context. Alternatively, if an allied health professional operates in a large area encompassing many villages, it may be less appealing to focus on a singular local initiative.

## Conclusion

In conclusion, we showed that the number of networks with allied health professionals increased during the DementiaNet project. Still, less than half of the DementiaNet networks included allied health professionals at follow-up, while the reasons for this are unknown. Therefore, qualitative exploration of possible barriers or facilitators for allied health involvement in these networks is needed. Further research on the effectiveness of allied health services is warranted to highlight the benefit of allied health inclusion in these networks.

## Abbreviations

PDCA, Plan-Do-Check-Act.
